# Information Exploration System for Sickle Cell Disease and Repurposing of Hydroxyfasudil

**DOI:** 10.1371/journal.pone.0065190

**Published:** 2013-06-10

**Authors:** Magbubah Essack, Aleksandar Radovanovic, Vladimir B. Bajic

**Affiliations:** Computer, Electrical and Mathematical Sciences and Engineering Division (CEMSE), Computational Bioscience Research Center (CBRC), King Abdullah University of Science and Technology (KAUST), Thuwal, Saudi Arabia; Beijing Institute of Genomics, Chinese Academy of Sciences, China

## Abstract

**Background:**

Sickle cell disease (SCD) is a fatal monogenic disorder with no effective cure and thus high rates of morbidity and sequelae. Efforts toward discovery of disease modifying drugs and curative strategies can be augmented by leveraging the plethora of information contained in available biomedical literature. To facilitate research in this direction we have developed a resource, Dragon Exploration System for Sickle Cell Disease (DESSCD) (http://cbrc.kaust.edu.sa/desscd/) that aims to promote the easy exploration of SCD-related data.

**Description:**

The Dragon Exploration System (DES), developed based on text mining and complemented by data mining, processed 419,612 MEDLINE abstracts retrieved from a PubMed query using SCD-related keywords. The processed SCD-related data has been made available via the DESSCD web query interface that enables: a/information retrieval using specified concepts, keywords and phrases, and b/the generation of inferred association networks and hypotheses. The usefulness of the system is demonstrated by: a/reproducing a known scientific fact, the “Sickle_Cell_Anemia–Hydroxyurea” association, and b/generating novel and plausible “Sickle_Cell_Anemia–Hydroxyfasudil” hypothesis. A PCT patent (PCT/US12/55042) has been filed for the latter drug repurposing for SCD treatment.

**Conclusion:**

We developed the DESSCD resource dedicated to exploration of text-mined and data-mined information about SCD. No similar SCD-related resource exists. Thus, we anticipate that DESSCD will serve as a valuable tool for physicians and researchers interested in SCD.

## Introduction

As a life-threatening monogenic disorder, Sickle cell disease (SCD) is the most common and is particularly common among people with sub-Saharan African, South American, Central American, Saudi Arabian, Indian, Turkish, Greek, and Italian ancestry [1]. The U.S. Centers for Disease Control and Prevention (CDC) website (http://www.cdc.gov/NCBDDD/sicklecell/data.html) states that:

- “SCD affects an estimated 70,000 to 100,000 Americans”
- “Sickle cell disease is a major public health concern. From 1989 through 1993, there was an average of 75,000 hospitalizations due to sickle cell disease in the United States, costing approximately $475 million.”

Currently, no cure or effective treatment exists for SCD. Simple interventions such as newborn screening for fetal hemoglobin [2] and the screening of prospective partners for abnormal hemoglobin genes have been implemented to significantly reduce mortality and incident rates, respectively [3]. Additionally, current research focuses on disease modifying drugs and curative strategies such as gene therapy [4] stem cell transplantation [5] and hemoglobin F (HbF) inducers [6], as these will probably have the best impact on SCD patients. Nonetheless, the sequelae and morbidity of the disease remains high. Efforts toward discovery of SCD modifying drugs can be augmented by leveraging the plethora of molecular and other information in published biomedical literature. We retrieved 419,612 SCD-related MEDLINE abstracts from PubMed limited to those published before 30/09/2012. Of these, 26% (108,227) were published in the last decade. This volume of biomedical information is far too big for an individual researcher(s) to process within a reasonable timeframe. Additionally, cross-data integration is difficult because molecular data exists in a variety of formats [7], [8]. Thus, the development of an integrated knowledgebase focused on SCD is attractive for researchers in this field. We have developed one such resource, Dragon Exploration System for Sickle Cell Disease (DESSCD) (http//cbrc.kaust.edu.sa/desscd/), based on the text mining approach and complemented by data mining.

DESSCD summarizes information form a large volume of raw data as it aims to automatically distill information, extract concepts, discover implicit links by association between the concepts, and generate hypotheses. This generation of hypotheses is known as ‘Text-Based Knowledge Discovery’ or ‘Literature Based Discovery (LBD)’ [9]. For example, Smalheiser and Swanson used text mining to correctly infer a link between Alzheimer’s disease and indomethacin, with the phrases ‘Indomethacin decreases plasma membrane fluidity in various cell types’ and ‘membrane fluidity is elevated in some patients with AD’, with ‘membrane fluidity’ being the connecting concept [9], [10]. Wren *et al*. also used LBD to infer a link between chlorpromazine and the development and/or progression of cardiac hypertrophy, ‘development and/or progression’ being the connecting concept. It was demonstrated that the progression of cardiac hypertrophy in rodent models is reduced with chlorpromazine treatment [11]. Natarajan *et al*. combined gene expression analysis and text mining of full-text journal articles to infer a relationship between invasiveness of the glioblastoma cell line and sphingosine 1-phosphate (S1P) [12]. It was demonstrated that S1P independently regulate glioblastoma cell invasiveness through urokinase-type plasminogen activator receptor and plasminogen activator inhibitor-1 expression [13].

Many such biomedical text mining tools that offer various functioning for LBD are available online. SciMiner (http://jdrf.neurology.med.umich.edu/SciMiner/), a functional analysis and literature mining tool that offers gene and protein identification using context specific analysis of MEDLINE abstracts and full texts articles [14]; MedlineRanker (http://cbdm.mdc-berlin.de/tools/Medlineranker), a text mining tool that enables ranking of MEDLINE entries without expert knowledge [15]; Polysearch (http://wishart.biology.ualberta.ca/polysearch/), a text mining tool that identifies relationships between tissues, cells, diseases, gene/protein names and drugs, whilst further highlighting and ranking informative literature [16]. More recently, topic-specific text mining tools in the biomedical field have become increasingly available online, such as those based on the Dragon Exploration System (DES) resources from OrionCell (http://www.orioncell.org). Examples are: 1/DESHCV (http://cbrc.kaust.edu.sa/deshcv/) that is useful for the exploration of Hepatitis C Virus related information [17], 2/DDEC (http://apps.sanbi.ac.za/ddec/) and DDOC (http://apps.sanbi.ac.za/ddoc/) which allows for the exploration of genes implicated in esophageal [18] and ovarian [19] cancers, respectively, 3/DDESC (http://apps.sanbi.ac.za/ddesc/) that provide comprehensive sodium channel related text mining information [20], 4/DESTAF (http://cbrc.kaust.edu.sa/destaf/) with information about toxins that affect fertility [21], DDPC (http://cbrc.kaust.edu/ddpc/) [22] that provides information on prostate cancer, etc.

To the best knowledge of the authors, no SCD-specific knowledgebase has ever been developed for the exploration of associations between biomedical SCD-related concepts. In this study, we present a novel DES-based biomedical text mining and data mining resource, Dragon Exploration System for Sickle Cell Disease (DESSCD). SCD-related PubMed abstracts were analyzed using concepts from the following dictionaries: “Human Genes and Proteins”, “Metabolites and Enzymes”, “Pathways”, “Chemicals with Pharmacological Effects”, “Human Anatomy Related Concepts” and “Disease Related Concepts”. These dictionaries have been cross-referenced to databases such as UNIPROT [23], KEGG Pathway [24], Entrez Gene [25] and REACTOME [26].

Researchers can explore DESSCD via a basic user query interface that allows for concept, keyword and phrase searches. DESSCD processed queries allow the user to: a/scrutinize processed PubMed abstracts with color labeled concepts from precompiled biomedical dictionaries, b/explore associations between concepts using the paired concept list, c/generate hypotheses that serve as the preliminary step in LBD, d/create networks of the relationship between the color labeled concepts and of the hypotheses generated, and e/download the “entity list” and “entity pairs list” for exploration of association between concepts. Additionally, the user is provided access to the downloadable manual, ‘A guide to using the Dragon Exploration System for Sickle Cell Disease (DESSCD)’. DESSCD is freely accessible to non-profit and academic users at http://www.cbrc.kaust.edu.sa/desscd/and will be updated biannually to incorporate newly published literature.

### Construction and Content

DESSCD is an integrated knowledgebase with a construction centered on the three-tier (layer) architecture (data, logic and presentation), the features and characteristics of which have been discussed elsewhere [18], [27].

DESSCD contains SCD-related biomedical data compiled based on MEDLINE abstracts retrieved from PubMed. We queried PubMed with the keyword expression: “(red blood cell) OR erythrocyte OR vaso-occlusive OR vasoocclusive OR antivasospastic OR vasospasm OR (sickle cell) OR sickle-cell OR anemia OR anemia” limited to abstracts published before 30/09/2012, 419,612 MEDLINE abstracts were retrieved. These abstracts (in the XML format) were then analyzed by DES. DES generated the DESSCD data files containing the biomedical concepts from the precompiled dictionaries as well as their links based on what has been found in the analyzed text.

Through DESSCD, one can explore information via the terms from six dictionaries: “Human Genes and Proteins”, “Chemicals with Pharmacological Effects”, “Human Anatomy Related Concepts”, “Disease Related Concepts”, “Pathways”, and “Metabolites and Enzymes”. These dictionaries include biomedical terminology with their variant names and symbols. In order to make the DESSCD we expanded dictionaries of “Chemicals with Pharmacological Effects”, “Human Anatomy Related Concepts” and “Disease Related Concepts” with SCD-related terms. As an example, the dictionary for “Human Genes and Proteins” includes the SCD-associated hemoglobin beta gene with its symbols, aliases and alternative names of genes and proteins. It should further be noted that concepts have been assigned to dictionaries based on their classification in literature. Thus, a concept such as docosapentaenoic acid (DPA) has been assigned to the “Chemicals with Pharmacological Effects” dictionary, even though it could have been assigned to the “Metabolites and Enzymes” dictionary. The explore option “Summary of results” allows for the exploration of the dictionary of choice and every concept within that dictionary has been linked to: a/the 419,612 MEDLINE abstracts, b/“EntrezGene ID”, “Uniprot ID”, “PathwayID”, “Pathway Name”, “Reactome” and “Reactome Name” were this information is available, c/other concepts that appear in abstracts of which a network can be generated, and d/a hypotheses generator to aid LBD. Additional exploring options include: a/the list of the DESSCD concepts “Entity list”, b/documents that contain the most DESSCD concepts “Frequency of documents”, c/the list of paired DESSCD concepts “Frequency of pairs” and d/the “Database statistics”. In addition, an “Entity list spreadsheet”, “Entity pair list spreadsheet” and “Database documentation” are downloadable.

One should note that systems for automated extraction of information are far from perfect. The common problem is that concepts identified are not always properly categorized. For example, a gene symbol could be wrongly categorized as an acronym for disease and vice versa. Also, dictionaries are never complete, so this will impact on the sensitivity of identifying named entity. Furthermore, the use of non-canonical names and symbols for genes, proteins and diseases is rather common in the biomedical field and is recognized as one of the underlying problem for automated analysis of text. This causes problems in correct identification of the named entities as some are missed and some are wrongly identified. In spite these problems, the enormous advantage of automated information extraction systems such as DESSCD, they compiled information from a very large volume of documents and other data sources that is impossible for a single user or group. In DESSCD the user can make an assessment of the correctness of the information when necessary, based on the context and the original information source and this makes a great practical utility for researchers.

## Results and Discussion

### Evaluating DESSCD by Reproducing a Known Fact as a Hypothesis

**Figure 1:**
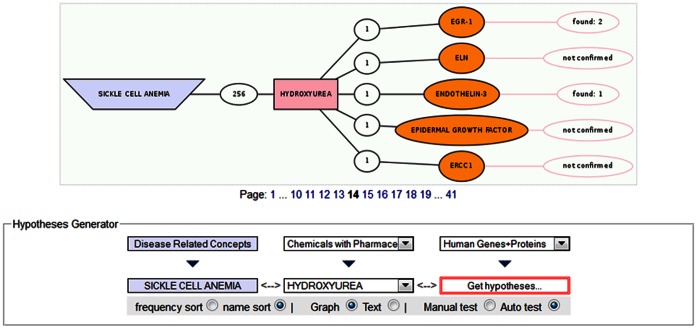
A graphical representation of DESSCD simulating a known fact, the “Sickle_Cell_Anemia–Hydroxyurea” association. SCA and Hydroxyurea were selected from the “Disease Related Concepts” and “Chemicals with Pharmacological Effects” dictionaries, respectively, whilst “Human Genes and Proteins” dictionary was selected as the target dictionary.

A number of LBD tools use the Swanson ABC model of “disjoint but complementary structures in biomedical literature to generate novel and plausible hypotheses” [9]. The performances of few of these LBD tools such as the DAD-system [28], Anni 2.0 [29], and the DES resource DESHCV [27] have been evaluated by simulating a confirmed scientific discovery. Another DES resource, DDESC also evaluated the performance of DES and reported that the precision and recall for identified concepts from “Human Genes and Proteins” dictionary was lower than the other dictionaries, with precision, recall and *F*-measure of 81.1%, 96.1% and 87,9%, respectively [20]. In this report, we assess the functioning of another DES resource, DESSCD by simulating a confirmed scientific fact, the “Sickle_Cell_Anemia-Hydroxyurea” association. To employ the open discovery approach we selected the explore option “Relations Summary”. We then changed the dictionary by selecting the dictionary “Disease Related Concepts”, clicked on “S”, scrolled to Sickle Cell Anemia (SCA) on page “51” and clicked on show hypothesis. In the “Hypotheses Generator”, Hydroxyurea in the “Chemicals with Pharmacological Effects” dictionary was hand selected as the connecting set of concept, while “Human Genes and Proteins” was selected as the target dictionary. Selecting “Auto test” followed by “Get Hypotheses” allowed the system to successfully generate hypotheses. The relationship between SCA and Hydroxyurea was confirmed with the retrieval of early growth response-1 (EGR-1) and endothelin-3 (ET-3) as the target concepts in the “Human Genes and Proteins” dictionary (Figure 1).

Hydroxyurea is currently being used to treat sickle cell anemia patients, as it is accepted that Hydroxyurea inhibits vaso-occlusive crisis via stimulation of fetal hemoglobin (HbF) and nitric oxide (NO) production in SCD [30]. Vaso-occlusive crisis is characterized by an increased adherence of haematocytes to the vascular endothelium. It has been shown that SCA patients exhibit elevated levels of vasoconstrictor, ET-3 and that Hydroxyurea therapy significantly decreases the release of ET-3 [31]. Similarly, leukotrienes are elevated in inflammatory diseases such as SCA and it has also been shown that EGR-1 attenuates elevated leukotriene in endothelial cells [32]. Moreover, EGR-1 levels were demonstrated to be significantly decreased in SCA patients compared to normal individuals and that Hydroxyurea therapy significantly increases EGR-1 levels [33]. Thus, the supporting literature verifies that DESSCD hypothesis generation is reasonable.

### Using DESSCD to Generate Hypotheses in Search of Potentially Novel SCD Drugs

**Figure 2:**
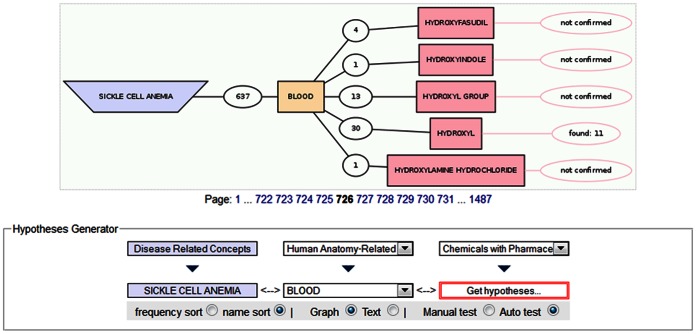
A graphical representation of the novel “Sickle_Cell_Anemia–Hydroxyfasudil” hypothesis. SCA and blood were selected from “Disease Related Concepts” and “Human Anatomy Related Concepts” dictionaries, respectively. Hydroxyfasudil was retrieved from the target dictionary, “Chemicals with Pharmacological Effects”.

A novel hypothesis was generated using DESSCD, proposing a relationship between SCA and a potential therapeutic agent, Hydroxyfasudil. We retrieved SCA from the “Disease Related Concepts” dictionary and in the “Hypotheses Generator”, the term blood in the “Human Anatomy Related Concepts” dictionary was hand selected as the connecting concept, while “Chemicals with Pharmacological Effects” was selected as the target dictionary. By doing this we retrieved Chemicals with Pharmacological Effects that have been implicated in blood-related diseases, other than SCA. Hypotheses were generated and tested automatically. An implicit relationship was inferred between SCA and Hydroxyfasudil, since these two concepts never occur together in any PubMed abstract implying a potentially novel association (Figure 2).

Thus, our study allowed for the identification of Hydroxyfasudil (http://www.drugbank.ca/drugs/DB04707), a recognized Rho-kinase inhibitor, as a potential novel SCA drug. Hydroxyfasudil has been demonstrated to attenuate pulmonary hypertension secondary to left ventricular dysfunction that is characterized by increased mean pulmonary arterial pressure, Rho-kinase activity, pulmonary arteriolar medial thickness, endothelin-1 (ET-1) and endothelial nitric oxide synthase (eNOS) concomitant with decreased cGMP and NO. Literature suggests that the effectiveness of Hydroxyurea for the treatment of SCD is a consequence of its ability to stimulate the NO-cyclic guanosine monophosphate (cGMP) signaling pathway to produce Hb [34]. Comparable to Hydroxyurea therapy, Hydroxyfasudil induces increased levels of eNOS, NO and cGMP that signify the possibility that Hydroxyfasudil may have the propensity to produce HbF via stimulation of the NO-cyclic guanosine monophosphate (cGMP) signaling pathway as well [35], but this potential of Hydroxyfasudil has not been experimentally proven.

Another aspect of interest is inflammation associated with SCD. An increased IL-6 levels have been associated with the pathophysiology of SCD and IL-6 has been demonstrated to play an important role in globin gene silencing [36]. Hydroxyfasudil has demonstrated the ability to reduce IL-6 levels after hypoxia/reoxygenation (H/R) injury in brain tissue [37], further increasing confidence in its ability to produce HbF. It should be noted here that IL-6 also acts as a pro-inflammatory cytokine in SCD, thus treatment with Hydroxyfasudil will additively reduce inflammation [37]. Alarmingly and contrary to this effect, it has been demonstrated that Hydroxyurea stimulates the expression of pro-inflammatory genes such as IL8, IL-6, IL1β, IL-1α, CCL20, CCL8, CCL5, and CCL2 in endothelial cells thereby enhancing the chronic inflammatory states in SCD [38]. Thus, Hydroxyfasudil demonstrates a beneficial and distinguishing effect of being able to reduce inflammation associated with SCD compared to Hydroxyurea.

Hydroxyurea has also been shown to alter the clinical symptoms of SCD by decreasing the levels of vasoconstrictors such as ET-1 [39] and ET-3 [31], and by affecting the degree of adherence of sickled erythrocytes and leukocytes through decreasing the levels of endothelial adhesion molecules such as sICAM-1, sVCAM-1, sSELE and sSELP [40]–[42]. Hydroxyfasudil has been shown to suppress IR injury-induced generation of ROS [43] and the levels of vasoconstrictor, ET-1 [35]; to reduce ICAM-1 expression in diabetes-induced microvascular damage [44], as well as to reduce ICAM-1, SELE and SELP thereby diminishing leukocyte – endothelial adhesion in colonic IR injury [45]. However, the effect of Hydroxyfasudil to vasoconstrictor ET-3 has not been experimentally determined. Hydroxyurea therapy has also been shown to affect erythrocyte–endothelial adhesion by reducing the expression of adhesion molecules such as CD36 and VLA-4 on the erythrocyte membrane [46]. Whether or not Hydroxyfasudil affects erythrocyte–endothelial or platelet–endothelial adhesion has not been experimentally determined. Similarly, whether or not Hydroxyurea therapy affects activators of the endothelium via HIF-1α or TF has not been experimentally determined. However, Hydroxyfasudil has been shown to reduce HIF-1α [47], TF [48] and NFκB [49].

Additionally, Hydroxyurea therapy decreased levels of inflammatory mediators such as GM-CSF [50] and TNF-α [51], and Hydroxyurea has been shown to increase levels of anti-inflammatory mediator IL-10 [51] attempting to reduce the inflammatory response but has not been effective. Similarly, Hydroxyfasudil has been shown to decrease pro-inflammatory mediators such as IL-1β [49] and TNF-α [49], and increase the anti-inflammatory mediator IL-10 [37]. Whether or not Hydroxyfasudil affects GM-CSF, IL-8 and IL-3 has not been experimentally evaluated.

Moreover, fibronectin, a glycoprotein encoded by FN1 gene, increases coagulation, thereby contributing to vaso-occlusion. Hydroxyurea has no demonstrable effects on the levels of FN1 [41], while Hydroxyfasudil reduces FN1 levels [52]. Thus, Hydroxyfasudil demonstrates a beneficial and distinguishing effect of being able to reduce coagulation associated with SCD compared to Hydroxyurea.

To summarize, Hydroxyfasudil demonstrates beneficial and distinguishing effects compared to Hydroxyurea that will a/better relieve vaso-occlusive crisis and b/reduce inflammation (Table 1). This link between SCA and the mechanism of action of Hydroxyfasudil draw attention to the possibility of combination therapy wherein the required HbF production can be induced by HbF inducers that are less toxic than Hydroxyurea when Hydroxyurea is used alone. To this effect, even Hydroxyurea could be used together with Hydroxyfasudil in dosages smaller than currently administered. This means that Hydroxyfasudil will compensate for known HbF inducers inability to effectively modulate vaso-occlusion and inflammation associated with the effective treatment of SCD. To the best of our knowledge, such a combination of Hydroxyfasudil with other drugs for treatment of SCD, which suggests the potential mode of activity of this combination of drugs, is now proposed for the first time.

**Table 1:** Effects demonstrated by Hydroxyfasudil compared to Hydroxyurea related treatment for SCD.

Molecules	Type of molecule	Expression statusin SCD	References	Effect of Hydroxyurea	Effect of Hydroxyfasudil
**ROS**	IR injury-related molecule	up	[61]	not known	decrease
**NO**	vasodilator	down	[62]	increase	increase
**ET-1**	vasoconstrictor	up	[63]	decrease	decrease
**ET-3**	vasoconstrictor	up	[31]	decrease	not known
**NFκB**	Activator of endothelial cells	up	[64]	decrease	decrease
**HIF-1**	Activator of endothelial cells	up	[65]	not known	decrease
**TF**	Activator of endothelial cells	up	[66]	not known	decrease
**eNOS**	Inhibitor of endothelial cell activation	down	[67]	increase	increase
**TNFα**	cytokine/inflammatory	up	[51]	decrease	decrease
**IL-1β**	cytokine/inflammatory	up	[68]	increase	decrease
**IL-8**	chemokine/inflammatory	up	[51]	increase	not known
**IL-6**	chemokine/inflammatory	up	[69]	increase	decrease
**IL-3**	inflammatory	up	[69]	increase	not known
**GM-CSF**	inflammatory	up	[50]	decrease	not known
**IL-10**	Anti-inflammatory	down	[51]	increase	increase
**VCAM-1**	adhesion	up	[70]	decrease	not known
**ICAM-1**	adhesion	up	[70]	decrease	decrease
**SELP**	adhesion	up	[70]	decrease	decrease
**SELE**	adhesion	up	[70]	decrease	decrease
**VLA-4**	adhesion	up	[46]	decrease	not known
**CD36**	adhesion	up	[46]	decrease	not known
**FN1**	coagulatory	up	[41]	no effect	decrease

Increase = increases protein levels; decrease = reduces protein levels; no effect = exert no effect on protein levels; not known = has not been experimentally determined.

Many HbF inducers such as Butyrate [53], Decitabine [53], Pomalidomide [54], Trichostatin A [55], Scriptaid [55], Suberoylanilide hydroxamic acid [55], Cucurbitacin D [56], Hydroxycarbamide [57], Rapamycin [58], 5-aza-cytidine [59] and apicidin [59] are being screened for the treatment of SCD. However, induction of HbF production via pathways dissimilar to that induced by Hydroxyurea therapy has rendered the inducers less effective than Hydroxyurea for the treatment of SCD [53]. Critically, our approach allowed us to determine that Hydroxyfasudil will reduce vaso-occlusion and inflammation associated with SCD whilst displaying the propensity to induce HbF production. One of the key points here is that Hydroxyurea increases HbF, while the same is not proved for Hydroxyfasudil. However, Hydroxyfasudil has been demonstrated to have other beneficial properties for SCD treatment that Hydroxyurea and other HbF inducers do not possess. Thus, we propose Hydroxyfasudil as a more appropriate drug for the treatment of SCD that we suggest to be used in a combination with inducers of HbF production. One of these inducers could be Hydroxyurea itself but in dosages smaller than currently being administered. Other HbF inducers have not been able to emulate the action of Hydroxyurea therapy as a modulator of vaso-occlusive crisis. Considering this shortcoming (i.e. the inability to modulate vaso-occlusive crisis) of the known HbF inducers screened for treating SCD, Hydroxyfasudil may be viewed as a complementary drug that will reduce the limitations of the less toxic HbF inducers. Taken together, this drug synergy approach will allow Hydroxyfasudil to be coupled with the most effective HbF inducer(s) that would ideally induce an enhancement of Hydroxyurea therapy whilst eliminating or reducing the associated side effects. A related PCT patent number PCT/US12/55042 covering Hydroxyfasudil as an alternative for treating SCD has been filed [60].

### Conclusion

We developed a user-friendly SCD-specific knowledgebase for the comprehensive probing for links between biomedical concepts. We have demonstrated that the system successfully reproduced the known “Sickle_Cell_Anemia–Hydroxyurea” link and generated novel and plausible “Sickle_Cell_Anemia–Hydroxyfasudil” hypothesis. The knowledgebase relies on the extracted textual data from the MEDLINE abstracts but as a future aim we plan to integrate information from full-text documents. We hope that this knowledgebase will serve as a valuable tool for physicians and researchers interested in SCD.
